# Insulin and Insulin-like Growth Factor 1 Signaling as a Modulator of MYC Expression in the Meibomian Gland

**DOI:** 10.3390/biomedicines14030578

**Published:** 2026-03-04

**Authors:** Cynthia Verling, Autumn Berlied, Cornelia Peterson

**Affiliations:** 1 Department of Comparative Pathobiology, Tufts University Cummings School of Veterinary Medicine, North Grafton, MA 01536, USA; cynthia.verling@tufts.edu (C.V.); autumn.berlied@tufts.edu (A.B.); 2 Tufts Center for Vision Research, Tufts Medical Center, Boston, MA 02111, USA

**Keywords:** meibocytes, Akt, sebaceous carcinoma, trofinetide, picropodophyllotoxin, demethylasterriquinone B1, autophagy

## Abstract

**Background/Objectives**: Sebaceous carcinomas (SebCAs) of the ocular adnexa, primarily arising from the Meibomian glands, are locally aggressive eyelid tumors with metastatic potential. Upregulation of the oncogene MYC has been demonstrated in SebCA, suggesting a role in tumor initiation and progression. In other epithelial tumors, the insulin and insulin-like growth factor (IGF) signaling (IIS) pathway has been implicated in stem cell renewal via MYC activation and stabilization. This study aimed to evaluate the effects of pharmacologic and genetic modulation of the IIS pathway on MYC expression in human Meibomian gland epithelial cells (HMGECs) and meibocytes of adult C57B6 mice. **Methods**: HMGECs were incubated with either IIS activators or inhibitors or were subject to transfection with either an *IGF1R* plasmid or siRNA before assessments of viability, proliferation, immunostaining, and MYC quantification were performed. Murine eyelids were treated topically with small-molecule IIS modulators prior to tissue harvest for histology, immunolabeling, and qPCR. **Results**: HMGECs treated with IIS activators demonstrated downregulated IGF1R and upregulated MYC expression, increased viability and proliferation, and reduced autophagy, while treatment with inhibitors yielded the inverse effects. Incubation with the selective insulin receptor agonist, demethylasterriquinone B1, yielded the most phenotypic variability. *IGF1R*-overexpressing HMGECs exhibited relative upregulation of both *Akt* and *MYC*. Murine eyelids treated with an IIS agonist demonstrated a more mesenchymal phenotype and significantly induced MYC expression. **Conclusions**: Collectively, these results suggest that the IIS pathway may represent a novel approach for regulating high MYC expression in SebCA.

## 1. Introduction

Meibomian glands (MGs) are specialized sebaceous glands within the tarsus of the eyelid that produce and secrete the lipid portion of the tear film [1,2]. Sebaceous carcinomas (SebCAs) of the ocular adnexa primarily arise from the human MG and are locally aggressive with high metastatic potential [1,3,4,5]. While the incidence of these tumors in the US is low, the age-adjusted incidence continues to significantly increase annually, with SebCAs now representing approximately 5% of all eyelid malignancies [6,7,8]. SebCAs present diagnostic challenges due to the mimicry of benign and inflammatory lesions such as chalazion and blepharoconjunctivitis [3,9,10]. Additional contributions to the aggressive phenotype include the propensity of this tumor to superficially metastasize by pagetoid spread to distant extraocular sites, ultimately leading to potential lymph node, lung, liver, bone or brain involvement [1,4,5]. There is an unmet need for precision therapies, as both the established surgical and non-surgical interventions, such as radiation or chemotherapy, often fail to limit local spread and tumor recurrence [1,4,5,9].

Protein upregulation and copy number gain of the c-*MYC* locus have been documented in a subset of SebCAs [11]. MYC oncoproteins affect critical cellular processes such as proliferation, protein synthesis, and the regulation of cell stemness [12,13]. MYC dysregulation has been noted in over 50% of human cancers, and its association with reduced patient survival and unfavorable prognoses is well-documented [12,14]. Despite the evidence of MYC’s role in oncogenesis, direct therapeutic targeting of MYC is challenged by the paucity of active drug binding sites [12,15]. Therefore, indirect modulation of MYC expression has been investigated as an alternative approach to developing potential novel cancer therapies [13,14,15,16].

In other carcinomas, such as breast and colon, the insulin/insulin-like growth factor-1 (IGF-1) signaling (IIS) pathway has been implicated in stem cell renewal via MYC activation and stabilization, resulting in expansion of more basaloid populations [13,17]. With respect to meibocytes, IGF-1-mediated stimulation of the PI3K/Akt pathway and subsequent increases in cell proliferation, differentiation and lipid accumulation have been demonstrated [2,18]. Notably, mutations in genes encoding proteins of the IIS pathway have been observed in SebCAs using next-generation sequencing (NGS) approaches, and MYC expression tended to be higher in SebCAs which harbored IIS-activating copy number alterations or mutations [11]. These findings support a connection between IIS dysregulation and MYC expression and suggest a collective role in promoting tumorigenesis of the MG [11,13,19,20].

The effect of dysregulating the IIS pathway, located upstream of MYC, in normal meibocytes has not been previously investigated. This study aimed to characterize how pharmacologic and genetic modulation of the IIS alters MYC expression and the oncogenic phenotype in non-neoplastic meibocytes, using both normal human Meibomian gland epithelial cells (HMGECs) and meibocytes of adult wildtype C57B6 mice.

## 2. Materials and Methods

### 2.1. In Vitro IIS Modulation

Early passage (P4-14) Human Meibomian Gland Epithelial Cells (HMGECs; ATCC, Manassas, VI, USA) were maintained in Keratinocyte Serum-Free media (KSFM; Gibco, Waltham, MA, USA) supplemented with Bovine Pituitary Extract (BPE; 50 µg/mL), human recombinant epidermal growth factor (EGF; 5 ng/mL) and normocin (100 µg/mL; InvivoGen, San Diego, CA, USA), and were incubated at 37 °C with 5% CO_2_ as previously described [21]. To assess the response of HMGECs to pharmacologic IIS modulation, cells were incubated with the IIS activators: trofinetide (synthetic IGF-1 analogue; Tocris Bioscience, Minneapolis, MN, USA), human recombinant insulin (endogenous IR agonist; Tocris Bioscience), and demethylasterriquinone B1 (DB1, selective IR activator; Tocris Bioscience); IIS inhibitors included picropodophyllotoxin (PPP, selective IGF1R inhibitor; Tocris Bioscience) and BMS 536914 (BMS, dual IR and IGF1R inhibitor; Tocris Bioscience). A small-molecule solvent (DMSO) was utilized as the vehicle control.

### 2.2. In Vitro Genetic Modulation of the IGF1R

HMGECs were either seeded into 6-well plates or 2-well chamber slides (*n* = 3 wells/condition) at a density of 2.1 × 10^5^ cells/well. At approximately 80% confluence, the cells were transfected with either 250 ng/well of *IGF1R* plasmid (plasmid #98344; Addgene, Watertown, MA, USA) or 25 pmol of *IGF1R* siRNA (s7211 Silencer Select; ThermoFisher, Waltham, MA, USA) in antibiotic-free Opti-MEM medium (ThermoFisher) using Lipofectamine 3000 (ThermoFisher) and standard protocols [22]. A scrambled plasmid (250 ng/well; plasmid #1864, Addgene) and 1x Stealth RNAi siRNA Negative Control Complexes (ThermoFisher) served as transfection controls. Transfected cells were maintained in Opti-MEM for five days prior to fixation or lysis.

### 2.3. In Vitro Viability Assessment

To assess cell viability, HMGECs were seeded into 96-well plates at 1000 cells/well. Cells were incubated in basal KSFM media for 24 h, after which they were treated with one of five IIS modulators using 2x serial dilutions or vehicle control (*n* = 3 wells/concentration/time point) for three days as follows: trofinetide (25, 50, or 100 nM), insulin (0.625, 1.25, or 2.5 μM), DB1 (1.25, 2.5, or 5.0 μM), PPP (0.313, 0.625, or 1.25 μM), and BMS (31.25, 62.5, or 125 nM). Initial concentration ranges were selected based on the vendor-provided pharmacodynamic documentation, FDA sponsor data, and a literature review of studies describing similar in vitro approaches [23,24,25,26,27,28,29,30]. A CyQuant MTT assay (ThermoFisher) was performed according to the manufacturer’s instructions, and the sample absorbance (optical density, OD) was read at 570 nm. Viable cell mass was determined using a standard curve.

### 2.4. Immunocytochemistry, Assessments of Proliferation, Autophagy, and Migration, and Evaluation of MYC Expression

To assess the response of HMGECs to IIS modulation, cells were seeded in 8-well chamber slides at a density of 2.0 × 10^4^ cells/well. Following a 24 h incubation with IIS modulators using trofinetide (100 nM), insulin (2.5 μM), DB1 (5.0 μM), PPP (1.25 μM), or BMS (125 nM), or a five day transfection as described above, chamber slides were fixed in 4% paraformaldehyde (PFA) and subjected to routine immunocytochemistry using an anti-c-MYC antibody (catalog #5605; Cell Signaling Technology, Danvers, MA, USA) diluted 1:400, an anti-IGF1R antibody (catalog #bs-4985R; Bioss, Woburn, MA, USA) at a dilution of 1:100, an anti-IRS2 antibody (catalog #ab134101; Abcam, Waltham, MA, USA) at a dilution of 1:500, or an anti-mTOR antibody (catalog #2983; Cell Signaling Technology) at a dilution of 1:200 in 3% BSA + 2% normal goat serum in 1x PBS + 0.1% Tween (PBS-T) incubated for 3 h at room temperature (RT). Chamber slides were then incubated with goat anti-rabbit, Alexa Fluor 488 (catalog #A-11008; ThermoFisher) or goat anti-rabbit, Alexa Fluor 555 (catalog #A-27039; ThermoFisher) at a dilution of 1:1000 for 60 min at RT. ReadyProbes Reagent F-actin probes (catalog # R37112 and R37110; ThermoFisher) were diluted in wash buffer and incubated for 30 min at RT. Slides were mounted in Prolong Gold Antifade with DAPI solution (ThermoFisher).

Proliferation was evaluated following 24 h incubation with IIS modulators using the Click-iT Plus EdU (5-ethynyl-2′-deoxyuridine) Imaging Kit (ThermoFisher) per the manufacturer’s instructions and the addition of EdU (10 µM) for the last four hours of drug incubation. Quantification of EdU and Hoechst-positive cells was performed by counting the number of positive nuclei for each stain in a 20x field (*n* = 4 fields/condition), and the proliferation rate for each 20x field was calculated using the following equation:

% proliferation = (number of Edu-positive cells/number of Hoechst-positive cells) × 100.

Autophagy was then evaluated in IIS-modulated HMGECs. A 16 h incubation with chloroquine diphosphate (30 µM; ThermoFisher) was included as a positive control. Briefly, an anti-LC3 antibody (catalog #L10382; ThermoFisher) was diluted 1:2000 in 3% BSA + 2% normal goat serum in 1X PBS-T for 60 min at RT followed by goat anti-rabbit, Alexa Fluor 488 at a dilution of 1:1000 in 3% BSA + 2% normal goat serum in 1x PBS-T for 60 min at RT. ReadyProbes Reagent F-actin probe was diluted in wash buffer and incubated for 30 min at RT prior to mounting as described above.

To evaluate migratory potential and epithelial–mesenchymal transition (EMT) in response to IIS modulation, HMGECs were seeded into 6-well plates or 4-well chamber slides at 2.0 × 10^5^ and 2.2 × 10^4^ cells/well, respectively. The following day, cells were incubated with IIS modulators (*n* = 3 wells/condition) including trofinetide (100 nM), insulin (2.5 μM), DB1 (5.0 μM), PPP (1.25 μM), or BMS (125 nM), and a 1 mm scratch was subsequently made in the cellular monolayer. Phase contrast photomicrographs were obtained immediately after the scratch was made and at regular intervals thereafter (12, 24, 48 h). Image J software (v.1.54p; National Institutes of Health, Bethesda, MD, USA) was utilized to quantify the initial scratch areas, and the remaining wound area was calculated relative to the initial area using the following equation:

% wound area remaining = (wound area measured at subsequent time points/wound area measured at t_0_) × 100.

HMGECs seeded into chamber slides were subjected to the same IIS modulating and wound conditions; however, following 24 h slides were then fixed in 4% PFA and subjected to immunocytochemistry using an anti-SLUG antibody (catalog #PA5-73015; ThermoFisher) at a dilution of 1:50 in 3% BSA + 2% normal goat serum in 1X PBS-T for 3 h at RT. DAPI staining and mounting was performed as described above.

A SimpleStep ELISA Kit (catalog #ab323521; Abcam) was used per the manufacturer’s instructions to evaluate c-MYC expression following 24 h incubation with IIS modulators (*n* = 3 wells/condition). Cell lysates were diluted to 150 µg/mL, the sample absorbance (OD) was read at 450 nm, and the c-MYC concentration was determined using the standard curve.

### 2.5. In Vivo IIS Modulation

Study approval was obtained from the Institutional Animal Care and Use Committee (IACUC) of Tufts University (protocol #G2023-01). Experiment planning and execution of protocols were conducted in adherence with the guidelines established by the Association for Research in Vision and Ophthalmology (ARVO) Statement for the Use of Animals in Ophthalmic and Vision Research. Reporting of in vivo experimentation information in this report are in compliance with the ARRIVE guidelines 2.0 for reporting animal research [31]. All mice utilized in this study were housed in a NexGen (Allentown, NJ, USA) individually vented caging system (*n* ≤ 5 adults/cage) within a temperature (20.0–22.2 °C; monitored and measured in Fahrenheit), humidity (30–70%) and 12 h day–night light-controlled environment with water and irradiated Teklad Global 18% Rodent Diet (Inotive, Indianapolis, IN, USA) ad libitum. Breeding females and weanlings were additionally supplied with supportive DietGel GEM dietary supplement (Clear H_2_O, Westbrook, ME, USA) to meet the nutritional demands in the gestational and peri-weaning periods.

Adult (P35-80) male and female C57B6 mice (strain #000664; The Jackson Laboratory, Bar Harbor, ME, USA) were subject to topical administration of IIS modulators as follows: IIS activation was achieved through once daily unilateral topical application of trofinetide (30 mg/kg/day) dissolved in DMSO to the eyelids and periocular skin for three consecutive days [32]. Antagonism of the IGF1R was achieved through once daily unilateral topical application of PPP (20 mg/kg/day) dissolved in DMSO to the eyelids and periocular skin for three consecutive days [33]. Vehicle-treated (DMSO) contralateral eyes served as controls. Mice were humanely euthanized 24 h after the last dose of respective IIS modulator. Eyelids with periocular skin and intact globes were harvested and were either formalin-fixed and paraffin-embedded (FFPE) or were homogenized for subsequent protein and RNA extraction.

### 2.6. Murine Meibomian Gland Morphologic Assessment and Immunohistochemistry

Hematoxylin and eosin (H&E)-stained whole mount sections of FFPE eyelid tissue harvested from IIS-modulated mice (*n* = 6) were used to visualize the morphology of the MGs. In situ protein expression was evaluated using either routine chromogenic immunohistochemistry or fluorescent immunohistochemistry and standard techniques. Antigen retrieval was performed using a sodium citrate buffer at 95 °C for 20 min. MYC expression was evaluated using the HRP-DAB IHC Detection Kit (Abcam) and an anti-c-MYC (Y69, Abcam) antibody at a dilution of 1:50 for 60 min at 37 °C. Proliferation was evaluated using an anti-Ki67 (catalog #ab15580; Abcam) at a dilution of 1:150 for 30 min at RT followed by the HRP-DAB IHC Detection Kit. Quantification of both Ki67-positive and -negative (hematoxylin stained only) cells was performed by counting the number of positive nuclei for each stain in three 20x fields/eyelid, and the proliferation rate for each 20x field was calculated using the following equation:

% proliferation = (number of Ki67-positive cells/number of total nucleated cells) × 100.

Murine thymic lymphoma was used as a positive control for MYC and Ki67. Epithelial and mesenchymal features were evaluated using an anti-E-cadherin antibody (catalog #PA5-85088; ThermoFisher) at a dilution of 1:250 and an anti-vimentin antibody (catalog #MA5-11883; ThermoFisher) at a dilution of 1:200 in 3% BSA + 2% normal goat serum in 1x PBS-T incubated overnight at 4 °C. Slides were then incubated with goat anti-rabbit, Alexa Fluor 488 and goat anti-mouse, Alexa Fluor 555 (catalog #A-21424; ThermoFisher) at a dilution of 1:1000 with 3% BSA + 2% normal goat serum in 1X PBS-T for 60 min at RT. DAPI staining and mounting was performed as described above.

### 2.7. Immunoblotting

Protein was isolated from homogenized murine eyelid tissue (*n* = 6 mice) using RIPA buffer containing phosphatase and protease inhibitor cocktails (ThermoFisher). Protein quantification was performed using a Pierce BSA. Normalized proteins were subject to SDS-PAGE using Mini-PROTEAN TGX Precast gels (Bio-Rad Laboratories Inc., Hercules, CA, USA) before electric transfer to PVDF membranes. Membranes were blocked with 5% BSA in tris-buffered saline (TBS) containing 0.01% tween-20 (TBS-T) for 60 min at RT before incubation with primary antibodies diluted in 1:1000 in 5% BSA in TBS-T overnight at 4 °C with the following antibodies: β-actin (catalog #PA1-183-HRP; ThermoFisher), c-MYC (catalog #5605; Cell Signaling Technology), IGF1Rß (catalog #3027; Cell Signaling Technology), and Akt (catalog #4691 Cell Signaling Technology). Membranes were incubated with a secondary antibody (donkey anti-rabbit HRP, catalog #31458; ThermoFisher) diluted 1:1000 in 5% BSA in TBS-T for 60 min at RT. Membranes were then visualized with enhanced chemiluminescence and a LI-COR Odyssey imaging system (LICORbio; Lincoln, NE, USA). Using Image J software densitometry was performed, and protein expression was normalized to β-actin.

### 2.8. RNA Isolation, Reverse Transcription, and Quantitative PCR (qPCR)

RNA was isolated from either HMGECs (*n* = 3 wells/condition) or homogenized murine eyelid tissue (*n* = 6 mice) using an RNeasy Kit (catalog #74104; Qiagen, Germantown, MD, USA). RNA concentration was determined using a NanoDrop spectrophotometer (ThermoFisher), and 500 ng of RNA was utilized for all subsequent reverse transcription reactions using a SuperScript VILO Kit (ThermoFisher). Quantitative PCR (qPCR) was performed using TaqMan Gene Expression Assays and QuantStudio 3 System (ThermoFisher). Expression of *IGF1R*, *Akt*, *MYC*, *CCDN2*, *LDHA*, *FASN*, and *ODC1* were quantified using 2^−ΔΔcT^ normalized to polR2α using predesigned probes: Hs0060956_m1 (human *IGF1R*), Mm00802831_m1 (murine *IGF1R*), Hs00982883_m1 (human *Akt1*), Mm01331626_m1 (murine *Akt*), Hs00153380_m1 (human *CCDN2)*, Hs01378790_g1 (human *LDHA*), Hs01005622_m1 (human *FASN*), Hs00159739_m1 (human *ODC1*), Hs00153408_m1 (human *MYC*), Mm00487804_m1 (murine *MYC*), Hs00172187_m1 (human *polR2α*), and Mm01309448_m1 (murine *polR2α*) (ThermoFisher).

### 2.9. Statistical Analyses

The distribution of each dataset was assessed for normality using the Shapiro–Wilk test prior to performing subsequent analyses. Statistical differences in proliferation rates, c-MYC concentration, and relative protein and transcript expression were assessed using one-way ANOVAs with Dunnett’s post hoc tests for multiple comparisons relative to respective controls. Viable cell mass and percentage of remaining wound area were evaluated using a two-way ANOVA with Dunnett’s post hoc tests for multiple comparisons relative to DMSO controls. All statistical analyses were performed using GraphPad (v. 10.6.1; San Diego, CA, USA) (α = 0.05).

## 3. Results

### 3.1. Variability in Cell Viability with Small-Molecule IIS Modulation In Vitro

**Figure 1:**
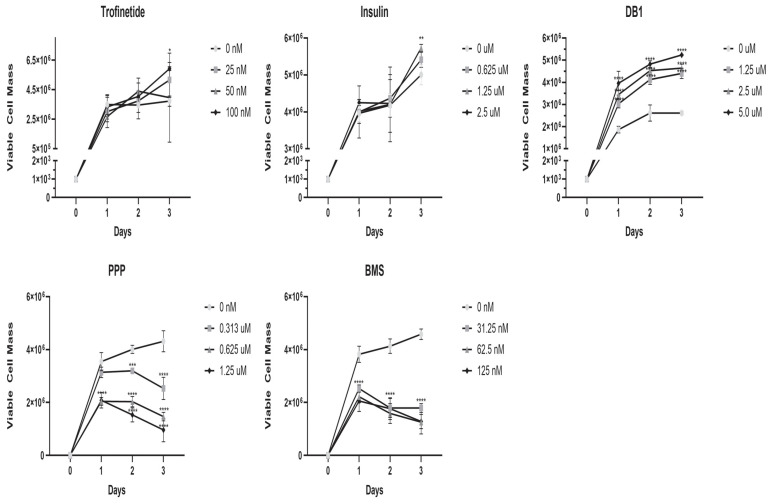
Impact of IIS modulation on HMGEC viability. An MTT assay was performed to assess cell viability of HMGECs in response to five different pharmacological IIS modulators each at three different concentrations (*n* = 3 wells/concentration/time point) over three days. HMGECs treated with IIS activators (top) exhibited increases in viable cell mass over time and a significant dose-dependent increase in viability in DB1-treated cells, while dose-dependent decreases were observed following pharmacologic inhibition (PPP, BMS-treated) of the IIS pathway (bottom). * *p* < 0.05, ** *p* < 0.01, *** *p* < 0.001, **** *p* ≤ 0.0001.

An MTT assay was performed to measure HMGEC viability following a three-day incubation with three concentrations (two-fold serial dilutions) of each of the five small-molecule IIS modulators evaluated. HMGECs treated with IIS activators such as trofinetide (*p* = 0.4115) and insulin (*p* = 0.8087) demonstrated expected increases in viable cell mass over time, independent of concentration, while HMGECs treated with DB1 exhibited a significant (*p* ≤ 0.0001) dose-dependent increase in viable cell mass, as shown in Figure 1. Conversely, HMGECs treated with IIS inhibitors, such as PPP (*p* ≤ 0.0001) and BMS (*p* ≤ 0.0001) exhibited a significant dose-dependent decrease in viable cell mass.

**Figure 2:**
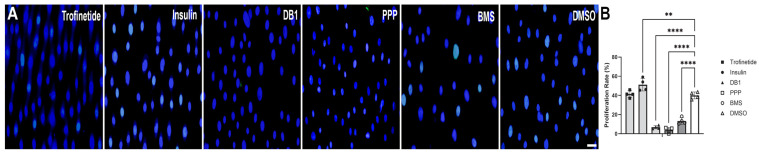
Proliferative responses to IIS modulation in vitro. (**A**) Fewer Edu (Alexa Fluor 488)-positive nuclei were observed in IIS-inhibited cells (yellow green in merged image). Hoechst (blue). Scale bar: 25 µm. (**B**) Proliferation was significantly increased following 24 h incubation with insulin (2.5 µM). Treatment with DB1 (5 µM), PPP (1.25 µM), and BMS (125 nM) significantly impaired proliferative responses. ** *p* < 0.01, **** *p* ≤ 0.0001.

Evaluation of cellular proliferative responses to 24 h of IIS modulation was determined using EdU immunolabeling (Figure 2A). HMGECs demonstrated significant reductions in proliferation rate following treatment with both IIS inhibitors (PPP: 3.8 ± 2.8%, *p* ≤ 0.0001; BMS: 13.3 ± 3.5%, *p* ≤ 0.0001) when compared to the DMSO control (40.0 ± 3.6%; Figure 2B). A significant reduction in proliferation was also observed in DB1-treated cells (6.8 ± 1.7%, *p* ≤ 0.0001). Proliferation was significantly increased in response only to the IIS activator insulin (51.2 ± 6.6%; *p* = 0.0032), while proliferation in trofinetide-treated cells (41.3 ± 3.6%) was not significantly different from DMSO controls (*p* = 0.9815).

**Figure 3:**
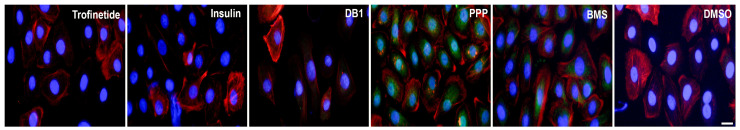
IIS regulation of autophagy. HMGECs treated with IIS inhibitors (PPP and BMS) exhibited increased autophagy (LC3 expression: Alexa Fluor 488) when compared to IIS activators (trofinetide, insulin, DB1) and vehicle control (DMSO). Phalloidin (Alexa Fluor 555), DAPI (blue). Scale bar: 10 µm.

An LC3 assay was subsequently performed to evaluate for autophagy in response to a 24 h incubation with trofinetide (100 nM), insulin (2.5 μM), DB1 (5 µM), PPP (1.25 µM), BMS (125 µM), or vehicle control (DMSO). HMGECs treated with IIS inhibitors demonstrated increased LC3 expression, localizing to perinuclear vacuoles relative to IIS-activated and control cells (Figure 3).

### 3.2. Differential MYC Expression Following Pharmacologic Modulation of the IIS Pathway In Vitro

**Figure 4:**
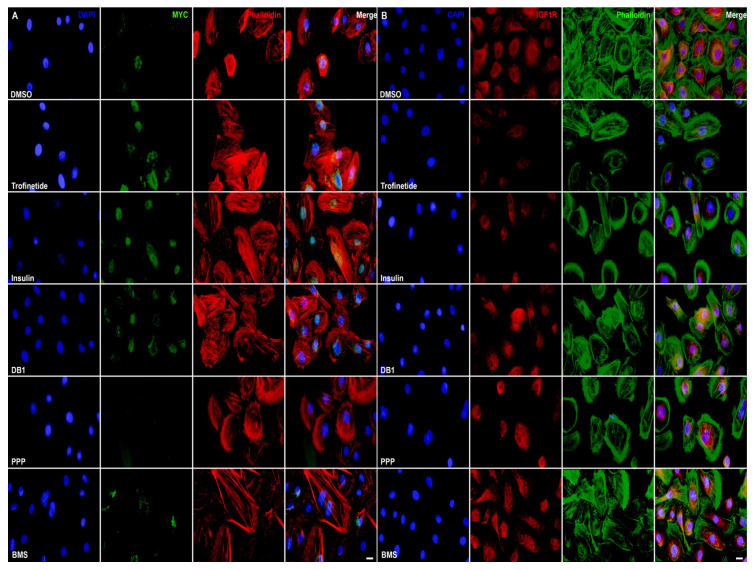
IIS modulation of MYC and IGF1R in vitro. HMGECs were incubated with trofinetide (100 nM), insulin (2.5 µM), DB1 (5.0 µM), PPP (1.25 µM), BMS (125 nM) and DMSO for 24 h. (**A**) Induction of MYC (Alexa Fluor 488) was demonstrated in HMGECs following incubation with IIS activators (trofinetide, insulin, and DB1) relative to vehicle control (DMSO). Phalloidin: Alexa Fluor 555. (**B**) IGF1R (Alexa Fluor 555) was downregulated in HMGECs following incubation with IIS activators (trofinetide and insulin) relative to vehicle control (DMSO). Phalloidin: Alexa Fluor 488. DAPI (blue). Scale bar: 10 µm.

To determine the effects of IIS activation or inhibition on MYC and IGF1R expression, HMGECs were seeded in an 8-well chamber slide and incubated with trofinetide, insulin, DB1, PPP, BMS, and DMSO. IIS-activated HMGECs exhibited concurrent MYC upregulation (Figure 4A) and IGF1R downregulation (Figure 4B) relative to vehicle control, while the inverse was observed in IIS-inhibited cells.

**Figure 5:**
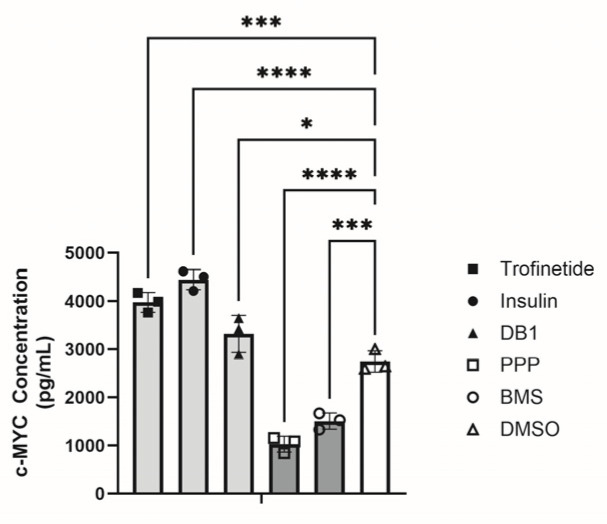
Quantification of IIS-modulated MYC expression. Assessment of c-MYC protein concentration by ELISA demonstrated significant increases in MYC concentration following treatment with IIS activators (trofinetide, insulin, and DB1; light grey) and significant decreases in MYC concentration in response to IIS inhibitors (PPP, BMS; dark grey) relative to vehicle control (DMSO; white). * *p* < 0.05, *** *p* < 0.001, **** *p* ≤ 0.0001.

To more quantitatively assess alterations in MYC expression resulting from 24 h of IIS modulation, MYC concentration was measured using an ELISA. MYC was significantly induced in response to IIS activation with trofinetide (3975.8 ± 206.1 pg/mL; *p* ≤ 0.0001, Figure 5), insulin (4448.4 ± 209.9 pg/mL; *p* ≤ 0.0001), and DB1 (3321.5 ± 385.9 pg/mL; *p* = 0.0451). MYC concentration was significantly attenuated following IIS inhibition relative to DMSO controls (PPP: 1029.8 ± 236.4 pg/mL; *p* ≤ 0.0001; BMS: 1508.1 ± 170.0 pg/mL; *p* = 0.0001; DMSO: 2745.4 ± 222.7 pg/mL).

To further characterize the downstream effects of IIS modulation on HMGECs, the expression of two critical IIS effectors, Insulin Receptor Substrate-2 (IRS-2) and mechanistic target of rapamycin (mTOR), was evaluated using immunocytochemistry (Supplementary Figure S1). Both IRS-2 and mTOR expression were induced in response to IIS activation, while IIS inhibitors suppressed expression relative to vehicle control.

**Figure 6:**
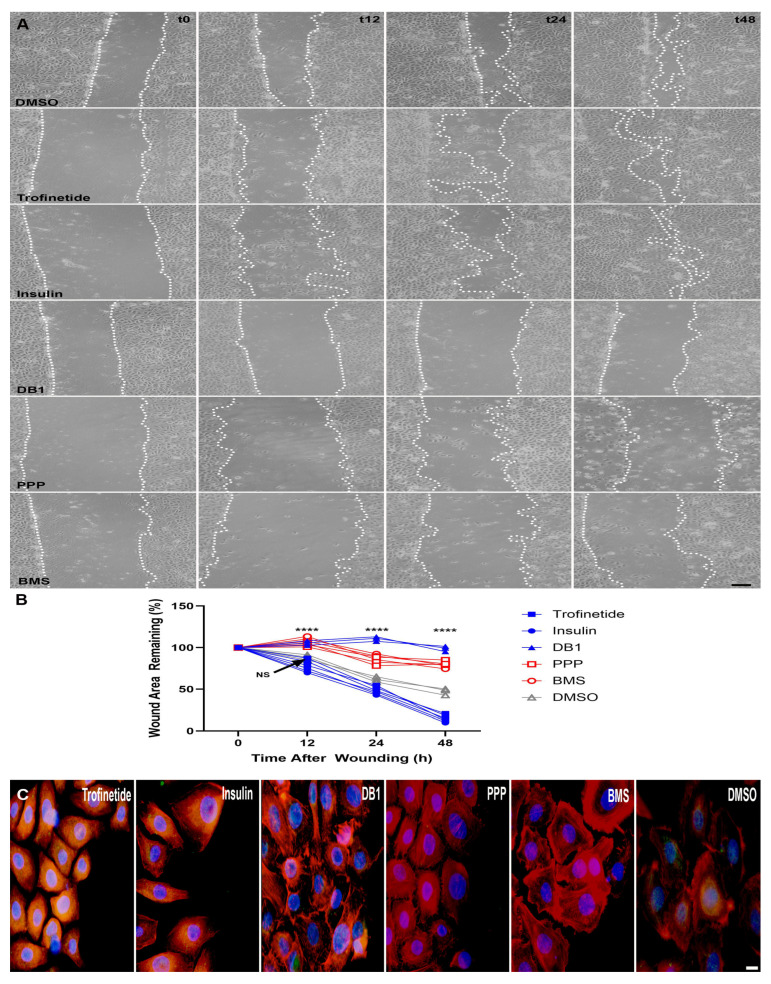
IIS modulation of migratory capacity in vitro. (**A**) Representative phase contrast photomicrographs of HMGECs incubated with IIS modulators over time. Remaining wound area annotated with a white dotted line. Scale bar: 50 µm. (**B**) The percentage of wound area remaining over time in response to IIS activators (blue) was significantly reduced relative to vehicle controls (grey), while IIS inhibitors (red) significantly impaired wound restoration. NS: not significant; **** *p* ≤ 0.0001. (**C**) SLUG (Alexa Fluor 488) was upregulated in HMGECs following incubation with IIS activators (trofinetide and insulin) relative to vehicle control (DMSO). Phalloidin: Alexa Fluor 555. DAPI (blue). Scale bar: 10 µm.

To assess the effects of IIS modulation on the migratory capacity of HMGECs, both scratch assays and immunostaining for the EMT marker, SLUG were performed. Incubation with all IIS modulators across all time points resulted in significantly different wound restoration with the exception only of trofinetide (83.0 ± 4.4% wound area remaining) vs. vehicle-treated (88.4 ± 3.1% wound area remaining) HMGECs (*p* = 0.0989) at the 12 h time point (Figure 6). IIS activators (trofinetide and insulin) yielded significantly greater wound restoration, with insulin treatment producing the greatest extent of monolayer reconstitution by 48 h (12.4 ± 2.7% wound area remaining). IIS inhibitors (PPP and BMS) demonstrated significantly impaired wound healing relative to DMSO. DB1-treatement also impaired wound healing, with an expansion of the wound area observed at 12 (105.6 ± 2.8% wound area remaining) and 24 h (110.6 ± 2.8% wound area remaining) post-wounding relative to the initial wound area. SLUG expression was promoted in response to IIS activation relative to DMSO controls in HMGECs at the leading edge of the artificial wound when assessed at 24 h post-wounding. Rare and mild SLUG expression was observed in DB1-treated cells, and SLUG expression was not demonstrated following 24 h incubation with IIS inhibitors.

**Figure 7:**
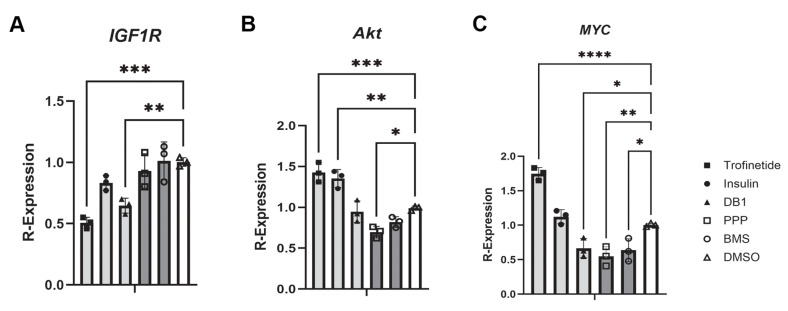
IIS modulation of transcript expression in vitro. (**A**) Relative expression of *IGF1R* was significantly downregulated in trofinetide and DB1-treated HMGECs. Relative (**B**) *Akt* and (**C**) *MYC* expression were significantly upregulated compared to DMSO controls (white) following treatment with the IIS activators (light grey) trofinetide and insulin, respectively, while the IIS inhibitors (dark grey) induced significant downregulation of *Akt* and *MYC*. * *p* < 0.05, ** *p* < 0.01, *** *p* < 0.001, **** *p* ≤ 0.0001. 2^−Δ∆cT^ was utilized to normalize target transcript expression to polR2α.

To quantitatively evaluate changes in relative gene expression in HMGECs following IIS modulation, cells were incubated with trofinetide (100 nM), insulin (2.5 µM), DB1 (5 µM), PPP (1.25 µM), BMS (125 nM), or DMSO for 24 h before being harvested and subject to routine qPCR. Interestingly, relative *IGF1R* expression was significantly reduced in trofinetide (0.51 ± 0.05; *p* = 0.0001) and DB1-treated cells (0.65 ± 0.06; *p* = 0.0025, Figure 7A). Trofinetide-treated cells demonstrated an induction in relative *Akt* and *MYC* expression, while DB1, PPP, and BMS-treated cells exhibited downregulation of *MYC* (Figure 7B,C).

To evaluate the effects of IIS modulation on the expression of canonical MYC targets in vitro, relative fatty acid synthase (*FASN*; lipid metabolism regulating enzyme), lactate dehydrogenase (*LDHA*: glycolytic enzyme), ornithine decarboxylase (*ODC1*: polyamine biosynthesizing enzyme), and cyclin D2 (*CCDN2*: cell cycle regulator) were quantified using routine qPCR. Relative *FASN* expression was significantly upregulated in response to both trofinetide (1.98 ± 0.17, *p* = 0.0079) and insulin treatment (3.7 ± 0.46, *p* ≤ 0.0001, Supplementary Figure S2). Incubation with trofinetide significantly induced relative *LDHA* expression (1.34 ± 0.06, *p* = 0.0001), while incubation with IIS inhibitors yielded significant suppression (PPP: 0.76 ± 0.07, *p* = 0.0019; BMS: 0.67 ± 0.06, *p* = 0.0001). Relative *ODC1* expression was significantly upregulated in response to IIS activation (trofinetide: 2.49 ± 0.34, *p* ≤ 0.0001; insulin: 2.23 ± 0.28, *p* ≤ 0.0001). IIS activation significantly promoted relative *CCDN2* expression (trofinetide: 2.73 ± 0.42, *p* ≤ 0.0001; insulin: 1.88 ± 0.29, *p* = 0.0011), while IIS inhibition results in significant downregulation (PPP: 0.46 ± 0.09, *p* = 0.0342; BMS: 0.50 ± 0.04, *p* = 0.0481).

### 3.3. Consequences of Genetic Modulation of IGF1R In Vitro

Following five-day transfections, *IGF1R*-overexpressing HMGECs exhibited an upregulation of both MYC and IGF1R relative to transfection controls, while both MYC and IGF1R expression was attenuated in *IGF1R*-silenced cells (Supplementary Figure S3). To quantitatively evaluate gene expression in *IGF1R*-modulated cells, qPCR was performed. HMGECs transfected with an *IGF1R* plasmid demonstrated significant increases in relative *IGF1R*, *Akt*, and *MYC* expression when compared to transfections controls (Supplementary Figure S4). *IGF1R* expression was efficiently and significantly overexpressed in *IGF1R* plasmid-transfected cells (1900.4 ± 351.1; *p* ≤ 0.0001), while relative *Akt* (9.1 ± 3.9; *p* = 0.0043) and *MYC* expression (11.1 ± 2.0; *p* ≤ 0.0001) were induced to an extent two orders of magnitude less.

### 3.4. Assessing Morphologic Changes Following Pharmacologic Modulation of the IIS In Vivo

**Figure 8:**
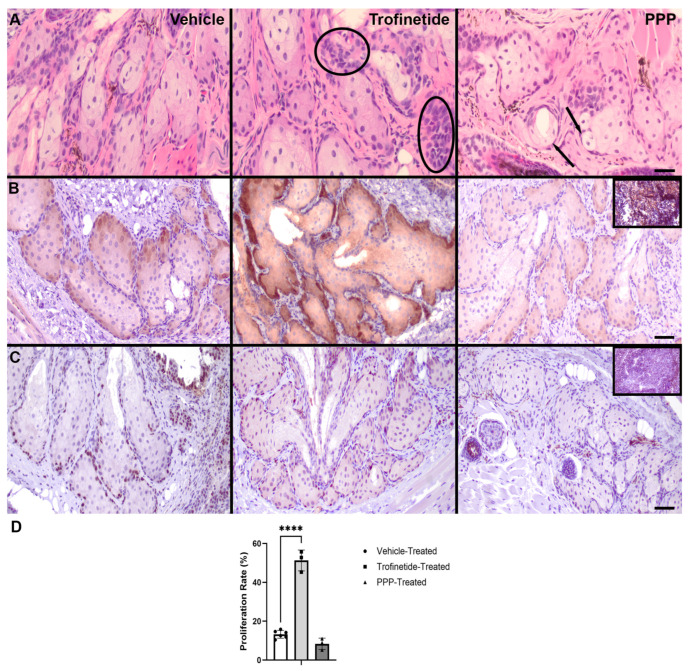
Morphologic features and altered MYC expression in the IIS-modulated murine Meibomian gland. (**A**) H&E-stained sections of FFPE murine eyelid. Trofinetide-treated MGs exhibited a mild expansion of the outer basal cells (circled regions). PPP-treated MGs demonstrated more sebaceous differentiation (arrows). Scale bar: 25 µm. (**B**) MYC-immunolabeled sections of FFPE murine eyelid. MGs obtained from eyelids subject to topical trofinetide for three days exhibited a robust induction of MYC expression (DAB: brown) in this same population of proliferative cells compared to vehicle and PPP-treated animals. Inset: murine thymic lymphoma, positive control. Scale bar: 50 µm. (**C**) Ki67-immunolabeled sections of FFPE murine eyelid. MGs obtained from eyelids subject to topical trofinetide for three days exhibited a robust induction of Ki67 expression (DAB) in this same population of proliferative cells and the more central, differentiated meibocytes, compared to vehicle and PPP-treated animals. Inset: murine thymic lymphoma, positive control. Scale bar: 50 µm. (**D**) The rate of proliferation was significantly increased in trofinetide-treated MGs compared to those from vehicle controls. **** *p* ≤ 0.0001.

To evaluate the effects of pharmacologic modulation of the IIS on the morphologic features and MYC expression of the murine MG, histology and immunohistochemistry for MYC expression was performed. Trofinetide-treated eyelids exhibited a mild increase in the abundance of the outer basaloid meibocytes, consistent with basal hyperplasia (Figure 8). MYC expression was robust in trofinetide-treated MGs relative to vehicle controls, with PPP-treated MGs exhibiting MYC downregulation. Ki67 was expressed in the outer basaloid cells of the vehicle-treated MG, with trofinetide treatment inducing expression in the basaloid and more differentiated meibocytes. The proliferation rate of trofinetide-treated MGs was significantly increased (51.3 ± 5.4%; *p* ≤ 0.0001) relative to vehicle controls (13.3 ± 2.0%). There was no significant difference in proliferation between PPP-treated MGs (8.4 ± 2.9%; *p* = 0.1113) and vehicle controls.

### 3.5. Alteration of Mesenchymal Features Resulting from Pharmacologic Modulation of the IIS In Vivo

**Figure 9:**
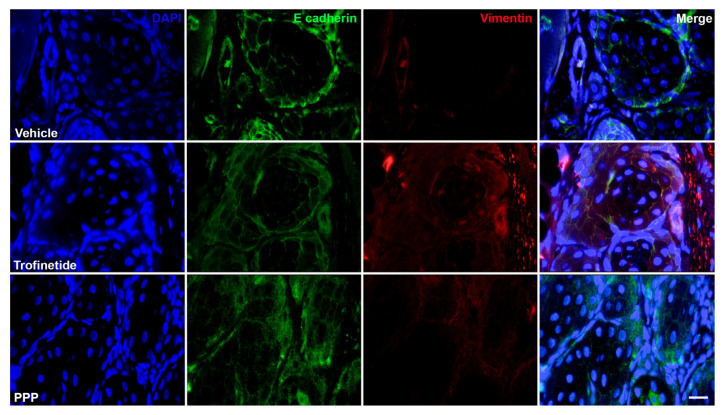
Evaluating the effect of IIS modulation on EMT in the murine Meibomian gland. Immunolabeled sections of FFPE murine eyelid. MGs obtained from eyelids treated with topical trofinetide for three days demonstrated increased vimentin (Alexa Fluor 555) expression relative to PPP-treated and vehicle control animals. E-cadherin (Alexa Fluor 488) expression was retained in all conditions, with mild attenuation in trofinetide-treated MGs. DAPI (blue). Scale bar: 25 µm.

To characterize the epithelial and mesenchymal features of the IIS-modulated murine MG, E-cadherin and vimentin co-labeling was performed. E-cadherin is a cell adhesion protein responsible for maintaining epithelial cell structure and integrity, while vimentin is an intermediate filament protein of the cytoskeleton and therefore plays a role in cell structure, movement and signaling and is typically absent in normal epithelial cells [34]. MGs treated with trofinetide exhibited an increase in the distribution of vimentin-positive meibocytes relative to the vehicle control and PPP-treated glands (Figure 9). While E cadherin expression was retained in trofinetide-treated MGs, mild attenuation relative to vehicle and PPP-treated eyelids was observed.

### 3.6. Protein and Gene Expression Profiles of IIS Modulation In Vivo

To determine the expression of IGF1Rß, MYC and Akt proteins in the IIS-modulated murine MG, homogenized tissue was subject to immunoblotting (Supplementary Figure S5A). Relative IGF1Rβ was not significantly different between treatment groups (vehicle: 1.0 ± 0.07; trofinetide: 0.64 ± 0.26, *p* = 0.1636; PPP: 1.46 ± 0.49, *p* = 0.0687). Relative expression of Akt (0.76 ± 0.05, *p* = 0.0031) and MYC (0.71 ± 0.16, *p* = 0.0065) were significantly downregulated following PPP treatment, and relative MYC expression (1.14 ± 0.05, *p* = 0.0317) was significantly increased in trofinetide-treated eyelids relative to contralateral controls (*Akt*: 1.02 ± 0.09; *MYC*: 0.95 ± 0.06, Supplementary Figure S5B).

**Figure 10:**
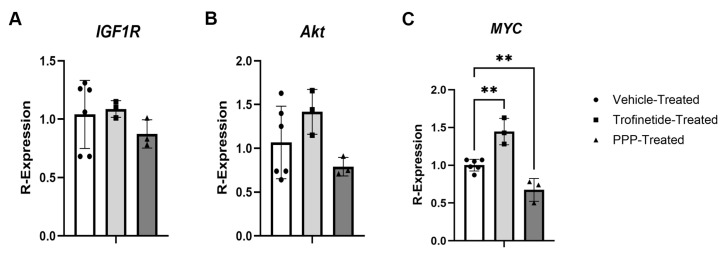
Differential transcript expression in the IIS-modulated murine Meibomian gland. Adult mice (*n* = 6) were topically treated with trofinetide, PPP, or vehicle, and eyelids were homogenized. There were no significant differences in relative (**A**) *IGF1R* or (**B**) *Akt* expression between treatment groups. (**C**) *MYC* expression was upregulated in trofinetide-treated murine MGs and downregulated in PPP-treated animals relative to respective contralateral vehicle-treated controls. ** *p* ≤ 0.01. 2^−Δ∆cT^ was utilized to normalize target transcript expression to polR2α.

To quantify the effects of IIS modulation on gene expression in vivo, homogenized murine MGs were subject to qPCR. No significant changes in either *IGF1R* or *Akt* expression were observed in trofinetide-treated (*IGF1R*: 1.1 ± 0.07, *p* = 0.9451; *Akt*: 1.4 ± 0.26, *p* = 0.2980) or PPP-treated eyelids (*IGF1R*: 0.87 ± 0.12, *p* = 0.5213; *Akt*: 0.79 ± 0.11, *p* = 0.4471) relative to contralateral vehicle controls (*IGF1R*: 1.04 ± 0.29; *Akt*: 1.07 ± 0.41, Figure 10A,B). Notably, relative *MYC* expression was significantly upregulated in trofinetide-treated MGs (1.45 ± 0.18, *p* = 0.0013) and downregulated in PPP-treated eyelids (0.67 ± 0.15, *p* = 0.0087) relative to respective contralateral vehicle-treated controls (1.00 ± 0.08; Figure 10C).

## 4. Discussion

The IIS pathway has a widely documented role in tumorigenesis and has been increasingly evaluated for therapeutic exploitation in the search for novel cancer therapies [13,17,20,35,36]. Recent research has offered a mechanistic association of the IIS pathway with MYC expression and stabilization, particularly via the IRS2/PI3K/GSK3b axis, to promote cancer stem cell self-renewal [13]. Although IGF-1 stimulation has been noted to activate the PI3K/Akt pathway, promote cellular proliferation, and exacerbate lipid production in HMGECs, its relationship with MYC expression has not been characterized [2,18]. NGSs of primary human ocular adnexal SebCAs have demonstrated copy number gains at the *MYC* locus, further supporting the role of this oncogenic driver in neoplastic meibocyte proliferation [11]. Additionally, activating mutations of *IGF2R* and *PIK3CA* have been documented in SebCA by other groups [11,37,38]. Here, we sought to characterize the meibocyte phenotypes, including MYC expression, in response to pharmacologic and genetic modulation of the IIS pathway. Receptor tyrosine kinases (RTKs) such as IGF1R, positioned proximally in the IIS pathway, served as initial targets for these modulatory approaches, with the expectation that future studies targeting more distal IIS components will identify which signaling elements most strongly regulate MYC.

The small molecules in this study were selected due to the IIS activation or inhibition potential for surface RTKs with consideration of the binding promiscuity of the insulin receptor (IR) family. The IGF1R is capable of binding multiple ligands including insulin, IGF-1 and IGF-2, albeit each with different affinities [13,19,39]. The IIS pathway is further complicated by both the structural homology of the IR and the IGF1R, which allow both insulin and IGF-1 to bind either receptor, and the potential for hybrid heterodimer generation, consisting of an α and ß subunit from each [17,19]. The presence of the hybrid receptor is important to consider in the context of cancer stem cell regulation, as it is thought that this receptor promiscuity might explain the correlation between hyperinsulinemia and an increased cancer risk reported in epidemiological studies [13,17,19]. Critically, the effects of the IIS modulators trofinetide, DB1, PPP, and BMS on MYC expression have not been previously reported despite the extensive literature supporting both their ability to modulate IIS and the subsequent impact of these pathways on downstream MYC regulation [25,40,41,42].

In this study we demonstrated that IIS pathway induction led to IGF1R suppression and downstream IRS-2, mTOR, and MYC upregulation in non-neoplastic meibocytes, similar to those reported across a spectrum of epithelial tumors [2,13]. Suppression of IGF1R expression in response to IIS activation may occur secondary to ligand-induced internalization via clathrin-mediated endocytosis (CME) and the subsequent reduction in receptor concentration at the cell surface membrane [43]. Both ligand-dependent ubiquitination, mediated by one of four E3 ligases, and subsequent routing for receptor degradation and clathrin-independent endocytosis, triggered when CME is oversaturated and less effective, result in the degradation and downregulation of the receptor via a caveolae/lipid raft pathway [35,36,43,44]. Treatment of HMGECs with IIS inhibitors yielded reciprocal effects. This pattern of differential expression was supported by approaches aimed at the genetic modulation of *IGF1R* in vitro, as *IGF1R*-overexpressing HMGECs exhibited relative upregulation of MYC expression, both at the protein and transcript level.

Cell viability, proliferation, autophagy, and migration were then assessed in IIS-modulated HMGECs. IIS-inhibited (PPP and BMS-treated) HMGECs demonstrated decreased viability, increased autophagy and/or compromised autophagosomal degradation, as evidenced by increased LC3 expression, and impaired migration when compared to IIS activators and vehicle control. These findings suggest that IIS pathway inhibition reduces MYC activity, triggering autophagy as a stress response in non-neoplastic meibocytes, with sustained blockade overwhelming this adaptive mechanism and ultimately driving cell death. The IIS pathway is generally autophagy-suppressive through mTORC1 activation and subsequent inhibition of ULK1 and other early autophagy components [45]. A relationship between dysregulated MYC expression and autophagy has been described in various neoplasms, with activation of MYC’s transcriptional program capable of repressing autophagy–lysosome genes, and MYC inhibition relieving this repression [46,47]. Additionally, inhibitors of the PITPNC1-MYC-mTOR axis, which promotes MYC stabilization and mTOR localization to lysosomes under physiologic conditions, downregulate MYC expression and induce autophagy [48]. This mechanism of MYC inhibition has been described as promoting anti-tumor effects in lung and pancreatic cancers [49]. The autophagy-inductive effects of PPP have been previously documented in multiple breast cancer cell lines [25,42].

MYC’s role in promoting cell migration and invasion through both its canonical transcriptional activity and noncanonical cytoplasmic roles, and its capacity to rewire cytoskeletal dynamics, adhesion, and EMT programs in both wound healing and cancer progression has been well-documented [50,51,52]. To interrogate the effects of IIS modulation on MYC-mediated migration in vitro, a scratch assay and immunolabeling for the EMT-regulating transcription factor SLUG were pursued [53]. IIS activation with trofinetide and insulin accelerated monolayer restoration and induced SLUG expression, suggesting that IIS induction of MYC expression may promote SLUG induction, a positive growth factor signaling–MYC–SLUG axis that has been described in stem cell renewal in other cell types [54].

Interestingly, our in vitro data demonstrated a significant dose-dependent increase in viability, concurrent significant decrease in proliferation and wound healing impairment, and no apparent alterations in autophagy in HMGECs incubated in DB1. This discrepancy between viability and proliferation may be due to a dissociation between metabolic and proliferative signaling pathways, in which DB1 exerts insulin-mimetic effects for metabolism through enhanced PI3K-Akt pathways while lacking canonical proliferative signaling targets [25,55]. This query also further highlights that utilizing MTT assays as a surrogate for direct cell counts can be challenged by the use of agents cable of promoting mitochondrial metabolism without altering cellular mitogenic drivers [56].

Next, we sought to characterize the effects of IIS modulation on MYC expression in the MG in vivo. Adult C57BL6 mice were utilized for these approaches as they offer a widely used and appropriate animal model due to their substantial genomic homology with humans, genetic uniformity which minimizes variability in experimental outcomes, and both MG structural features and lipid compositions that recapitulate those in humans [57,58]. These factors ensure that key pathways like IIS are as functionally analogous to humans as possible and support more reliable translation of drug effects from an animal model to human MG studies [59,60].

Murine eyelids treated with an IIS agonist demonstrated expansion of proliferative cell populations and increased Ki67 expression, primarily localizing to the more mitotically active outer basal cells, and a more mesenchymal phenotype. The mesenchymal features observed in trofinetide-treated MGs were demonstrated through concurrent but mild upregulation in vimentin with suppression of E-cadherin expression relative to PPP-treated and vehicle control glands. As discussed in our in vitro approaches, IGF-1 is a well-documented stimulator of EMT and cancer cell migration, suggesting that IIS induction may promote the early features of cellular migration and invasion, even in non-neoplastic meibocytes in vivo [35]. Interestingly, significant downregulation of Akt, and MYC protein expression in PPP-treated MGs was also demonstrated relative to vehicle control, and transcript profiling exhibited that relative *MYC* expression was significantly altered by IIS modulators. These in vivo data support the results of our initial in vitro studies. Additionally, our pilot study evaluating topical administration of PPP in vivo is consistent with prior studies which characterized it as an exception among IGF1R antagonists due to its ability to trigger IGF1R downregulation and inhibit downstream kinase activity [61]. PPP’s capacity to elicit biased signaling and induce tumor regression has been previously demonstrated in xenografted mice in response to systemic administration via intraperitoneal injections [28,36,62,63]. PPP’s specificity arises from its B-arrestin-biased agonism, which selectively inhibits IGF1R kinase activity at the substrate level, effectively blocking IGF1R autophosphorylation without competing for ATP or relying on classical G-protein coupled receptor (GPCR) signaling, a mechanism shared by both IGF1R and IR [28,61,64]. PPP may, therefore, represent a promising target for future research in the context of MG tumorigenesis for its described selectivity and our demonstration of topical tolerability in vivo.

We acknowledge several limitations to the current study. Primarily, the small-molecule IIS modulators utilized varied from those with selective IGF1R regulatory effects to those with capacity for dual targeting of IGF1R and IR, and the results were necessarily interpreted in context of the mechanism of action at the RTK. Numerous pathways converge on IIS pathway components, including other RTKs, such as ErbB receptor and platelet-derived growth factor receptor families, and GPCRs, making it challenging to determine whether modulators produce phenotypic effects solely through direct IIS regulation [65,66]. Dose optimization of some of the IIS modulators, most specifically DB1, represents an additional challenge, as the dose ranges most commonly documented in in vitro studies (3–10 µM) produced seemingly inconsistent phenotypic features in our cells [25,55,67,68]. However, we have attempted to provide possible rationale for these inconsistencies, and future studies to more specifically characterize these observations are warranted. Another consideration for our approach for IIS modulation in vivo, is our piloting of topical administration of PPP to the eyelids over a three-day treatment course, limiting the evaluation of IIS inhibition at later time points. However, this experiment will now allow future studies to more confidently develop experimental protocols defining the topical administration of PPP for longer durations and over different concentration ranges. Ongoing studies aim to evaluate pharmacologic targeting of IIS pathway kinases downstream of the RTKs (e.g., PI3K, Akt, mTOR) in the context of MYC modulation, while future in vivo studies could alternatively explore the consequences of MYC expression in conditional PI3K or Akt knockout models.

From a more clinical perspective, further evaluation of the use of IIS inhibitors as a means of modulating the MYC oncogene and suppressing proliferation and EMT is imperative. Interestingly, prior clinical trials have described disappointing outcomes or even the necessity to abandon IGF1R inhibitors due to dysregulated glucose metabolism, but, to date, the use of anti-IGF1R therapies has not been explored as an intervention for sebaceous malignancies [35,36,62,69,70]. A critical feature of studies describing off-target effects in breast, colorectal, prostate, ovarian and non-small cell lung cancer, is their systemic (i.e., intravenous) IGF1R targeting strategy [70]. Topical application, as would be indicated for ocular surface and adnexal tumors, would limit systemic absorption and circumvent these off-target complications.

## 5. Conclusions

This study sought to characterize the effect of IIS modulation on MYC expression in non-neoplastic meibocytes. We found that in vitro, IIS activation resulted in MYC upregulation and IGF1R downregulation, while incubation with IIS inhibitors, specifically PPP and BMS, demonstrated a significant dose-dependent decrease in viable cell mass and proliferation rate. The in vivo results complemented these findings and further revealed induction of MYC and promotion of mesenchymal features following IIS activation. Collectively, these results suggest that the IIS pathway may represent a novel approach for targeting high MYC expression in SebCA.

## Data Availability

The original contributions presented in the study are included in the article. Further inquiries can be directed to the corresponding author.

## Supplementary Materials

The following supporting information can be downloaded at: https://www.mdpi.com/article/10.3390/biomedicines14030578/s1, Figure S1: IIS modulation of IRS-2 and mTOR in vitro.; Figure S2: Effects of IIS modulation on canonical MYC targets in vitro; Figure S3: Effects of *IGF1R* modulation on MYC expression in vitro; Figure S4: IIS pathway modulation in *IGF1R*-overexpressing HMGECs; Figure S5: Differential protein expression in the IIS-modulated murine Meibomian gland.

## Abbreviations

The following abbreviations are used in this manuscript:

BMS	BMS 536914
CME	Clathrin-mediated endocytosis
DB1	Demethylasterriquinone B1
EMT	Epithelial-to-mesenchymal transition
GPCR	G-protein coupled receptor
H&E	Hematoxylin and eosin
HMGECs	Human Meibomian gland epithelial cells
IGF-1	Insulin-like growth factor 1
IGF1R	Insulin-like growth factor 1 receptor
IIS	Insulin and insulin-like growth factor 1 signaling
IR	Insulin receptor
IRS-2	Insulin receptor substrate-2
MG	Meibomian gland
mTOR	Mechanistic target of rapamycin
NGS	Next generation sequencing
OD	Optical density
PPP	Picropodophyllotoxin
RT	Room temperature
RTK	Receptor tyrosine kinase
SebCA	Sebaceous carcinoma
