# Supplementary material for: Insulin and Insulin-like Growth Factor 1 Signaling as a Modulator of MYC Expression in the Meibomian Gland
Source: Biomedicines. 2026 Mar 4;14(3):578. doi: 10.3390/biomedicines14030578 (PMC13024306; doi:10.3390/biomedicines14030578)
Supplement: Supplementary file 1 [file biomedicines-14-00578-s001.zip › biomedicines-4151556-supplementary.pdf]

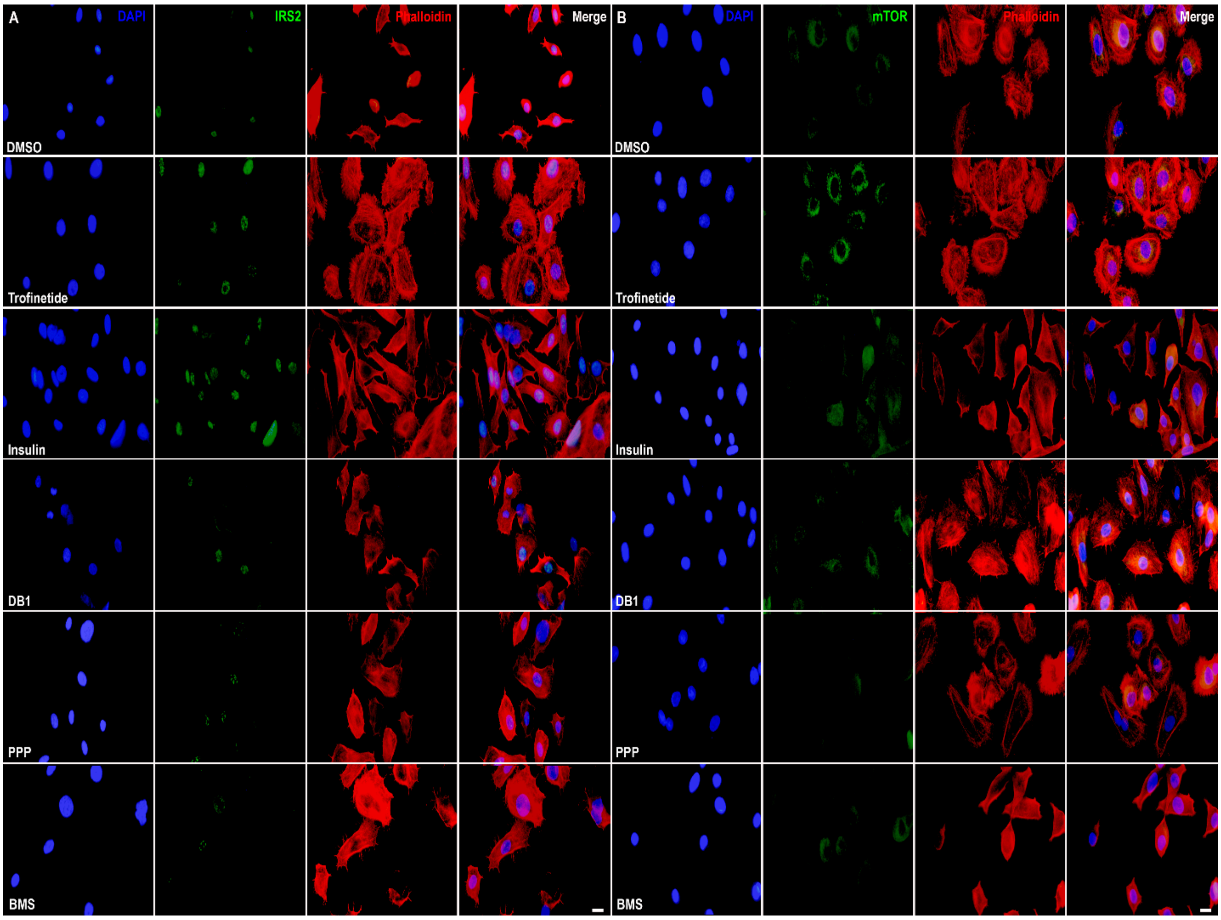

Supplementary Figure S1. IIS modulation of IRS-2 and mTOR *in vitro*. HMGECs were incubated with trofinetide (100 nM), insulin (2.5 μM), DB1 (5.0 μM), PPP (1.25 μM), BMS (125 nM) and DMSO for 24 hours. (**A**) IRS-2 (Alexa Fluor 488) was upregulated in HMGECs following incubation with IIS activators (trofinetide and insulin) relative to vehicle control (DMSO). Phalloidin: Alexa Fluor 555. Scale bar: 10 μm. (**B**) Induction of mTOR (Alexa Fluor 488) was demonstrated in HMGECs following incubation with IIS activators (trofinetide, insulin, and DB1) relative to vehicle control (DMSO), while IIS inhibitors (PPP and BMS) suppressed mTOR expression. Phalloidin: Alexa Fluor 555. DAPI (blue). Scale bar: 10 μm.

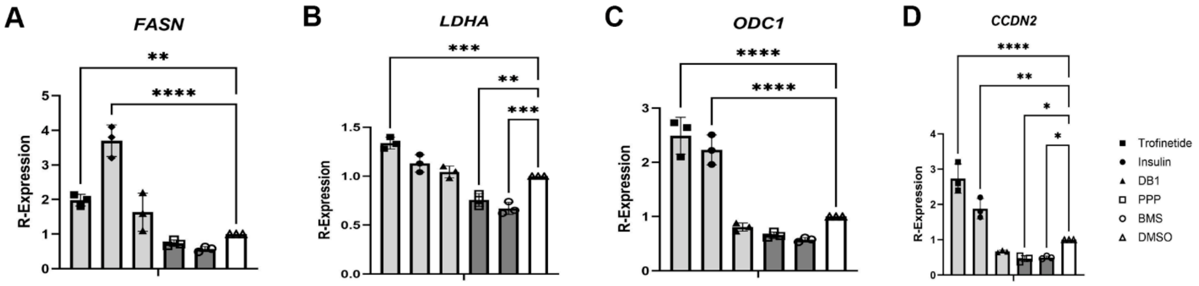

Supplementary Figure S2: Effects of IIS modulation on canonical MYC targets *in vitro*. (**A**) Relative expression of *FASN* was significantly upregulated in IIS activated (light grey) HMGECs incubated in trofinetide and insulin. (**B**) Relative *LDHA* expression was significantly induced in response to trofinetide treatment, while incubation with IIS inhibitors (dark grey) resulted in significant suppression. (**C**) Relative *ODC1* expression was significantly promoted in response to IIS activation with trofinetide and insulin. (**D**) *CCDN2* expression was significantly upregulated relative to DMSO controls (white) following treatment with the IIS activators trofinetide and insulin, respectively, while the IIS inhibitors induced significant downregulation. * $p < 0.05$, ** $p < 0.01$, *** $p < 0.001$, **** $p \leq 0.0001$. $2^{-\Delta\Delta cT}$ was utilized to normalize target transcript expression to polR2α.

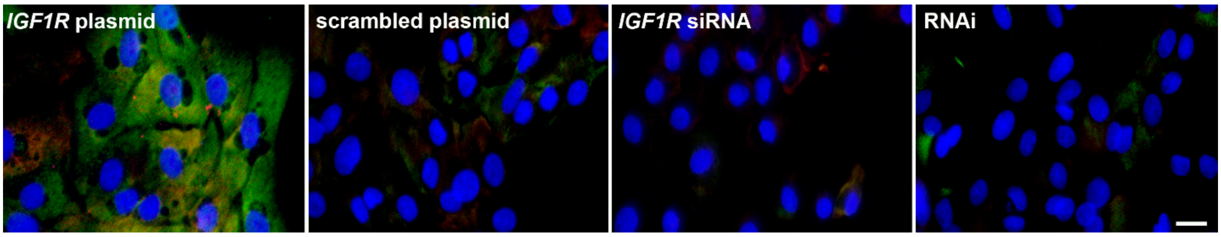

Supplementary Figure S3: Effects of *IGF1R* modulation on MYC expression *in vitro*. *IGF1R*-overexpressing HMGECs demonstrated an upregulation of both IGF1R (Alexa Fluor 488) and MYC (Alexa Fluor 555) relative to both transfection controls (scrambled plasmid and RNAi), and expression of both proteins was attenuated in *IGF1R*-silenced cells (siRNA). DAPI (blue) Scale bar: 10 μm.

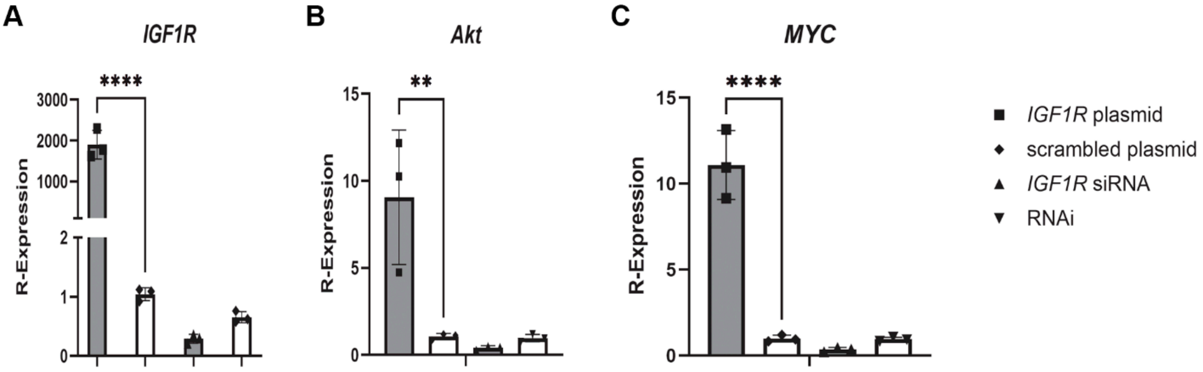

Supplementary Figure S4: IIS pathway modulation in *IGF1R*-overexpressing HMGECs. *IGF1R*-overexpressing HMGECs exhibited significant upregulation of *Akt* and *MYC*, while *IGF1R*-silenced cells demonstrated reduced relative expression. ** $p \leq 0.01$, **** $p \leq 0.0001$. $2^{-\Delta\Delta cT}$ was utilized to normalize target transcript expression to *polR2α.*

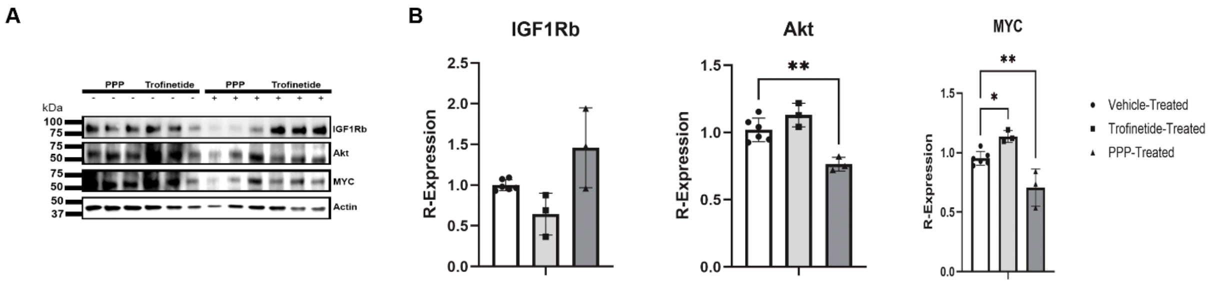

Supplementary Figure S5: **Differential protein expression in the IIS-modulated murine Meibomian gland.** Adult mice were topically treated with trofinetide, PPP, and vehicle for three days. Relative Akt and MYC expression were significantly attenuated in PPP-treated MGs relative to vehicle controls, and trofinetide treatment significantly upregulated relative MYC expression (normalized to actin expression). $^{*}p < 0.05$, $^{**}\ p \leq 0.01$.
